# What are the common downstream molecular events between alcoholic and nonalcoholic fatty liver?

**DOI:** 10.1186/s12944-024-02031-1

**Published:** 2024-02-08

**Authors:** Giovanni Tarantino, Vincenzo Citro

**Affiliations:** 1 Federico II University Medical School of Naples, Naples, 80131 Italy; 2 Department of General Medicine, Umberto I Hospital, Nocera Inferiore, SA 84014 Italy

**Keywords:** Hepatic steatosis, Alcoholic liver disease, Nonalcoholic fatty liver disease, Microbiome, Adipokines, Cytokeratins, Sirtuins, Spleen, Patatin-like phospholipase domain-containing proteins, miRs

## Abstract

Liver fat storage, also called hepatic steatosis, is increasingly common and represents a very frequent diagnosis in the medical field. Excess fat is not without consequences. In fact, hepatic steatosis contributes to the progression toward liver fibrosis. There are two main types of fatty liver disease, alcoholic fatty liver disease (AFLD) and nonalcoholic fatty liver disease (NAFLD). Although AFLD and NAFLD are similar in their initial morphological features, both conditions involve the same evolutive forms. Moreover, there are various common mechanisms underlying both diseases, including alcoholic liver disease and NAFLD, which are commonalities. In this Review, the authors explore similar downstream signaling events involved in the onset and progression of the two entities but not completely different entities, predominantly focusing on the gut microbiome. Downstream molecular events, such as the roles of sirtuins, cytokeratins, adipokines and others, should be considered. Finally, to complete the feature, some new tendencies in the therapeutic approach are presented.

## Introduction

Fatty liver or hepatic steatosis is increasingly diagnosed worldwide. In addition to histology, noninvasive techniques, such as ultrasound, computerized tomography magnetic resonance imaging and proton magnetic resonance spectroscopy, can be used to detect this disease [1]. A Japanese study showed that in 1989, the prevalence of fatty liver was 12.6%, which reached 30.3% in 1998, corresponding to a 2.4-fold increase over the previous rate; this figure was twice as high in males (26.0%) as in females (12.7%) [2]. Its main characteristic is the accumulation of triacylglycerol-rich macrovesicular and/or microvesicular lipid droplets within hepatocytes. This fat storage is not without consequences. In fact, hepatic steatosis contributes to the progression toward liver fibrosis [3]. There are two main types of fatty liver disease, alcoholic fatty liver disease (AFLD) and nonalcoholic fatty liver disease (NAFLD).

In recent years, there have been many discussions about the nomenclature of fatty liver disease among experts. In 2020, an international expert consensus recommended a new definition, MAFLD, which is metabolic dysfunction-associated fatty liver disease and represents the central concept of metabolic dysfunction [4]. The fatty liver disease terminology was also changed as of June 2023. MASLD has replaced NAFLD. To clarify the suggested nomenclature, MASLD can be diagnosed based on the presence of one of five cardiovascular risk factors, unlike MAFLD, which requires patients to have two of seven parameters of metabolic dysfunction [5]. The MASLD score is a very useful definition for lean patients with NAFLD [6]. Interestingly, patients who meet both the MASLD and alcohol-related fatty liver disease (ALD) criteria are categorized as having MetALD to include those with MASLD who consume greater amounts of alcohol per week (140 g/week and 210 g/week for females and males, respectively) [7]. Another necessary change in terminology was proposed, i.e., metabolic dysfunction-associated steatohepatitis (MASH), as a replacement for NASH to avoid the use of exclusionary confounder terms and potentially stigmatizing language [8]. A recent study revealed that the new nomenclature MASLD almost always confirmed NAFLD, while there were significant differences among MASLD, MetALD and MAFLD [9]. With respect to the various diagnostic terms used by patients, there were no substantial distinctions between “NAFLD”, “fatty liver disease”, “NASH”, or “MAFLD”. Furthermore, regarding the new nomenclature MASLD, the percentage of providers reporting “steatotic liver disease” as stigmatizing was low (14%) [10].

Finally, it has been confirmed that the discrepancies between NAFLD and MASLD criteria are negligible [11]. Based on such considerations and because most of the discussed studies, mainly preclinical studies, still illustrate the disease as NAFLD, we will employ such a definition throughout the manuscript, except when the pieces of research address MAFLD or MASLD.

AFLD and NAFLD exhibit the same progressive histological manifestations, i.e., steatohepatitis and cirrhosis, but the natural history of alcoholic liver disease (ALD) is often dissimilar from that of NAFLD in terms of clinical aspects [12]. In a registry-based study from 2003, the mortality of patients with a diagnosis of fatty liver increased 5.4-fold (95% CI 5.2–5.6) in patients with ALD and 2.6-fold (95% CI 2.4–2.9) in patients with NAFLD or unspecified fatty liver [13]. Currently, the scenarios are completely different. In fact, with high rates of obesity (likely one of the most important drivers) and type 2 diabetes mellitus (T2DM) among adult patients, which are associated with an aging population, NAFLD mortality is reported to steadily increase. Specifically, in the United States, the estimated incidence of hepatocarcinoma, the most severe sequela of NAFLD, will increase by 137% in 2030 [14]. According to 2017–2018 data from the National Health and Nutrition Examination Survey, more than 2 in 5 adults (42.4%) were obese, and approximately 1 in 11 adults (9.2%) had severe obesity [15]. The interaction of the changing food context combined with other environmental factors and genetic predispositions has created the current obesity pandemic, but the underlying mechanism has not been fully elucidated, resulting in many unanswered questions [16]. The fact that NAFLD and metabolic syndrome, with their main components, i.e., central obesity, abnormal glycemic values, dyslipidemia and hypertension, are intertwined is a finding dating back many years [17, 18]. However, the reciprocal effects of alcohol consumption and several metabolic syndrome criteria merit great attention in the risk stratification of severe liver complications and mortality [19, 20].

**Fig. 1:**
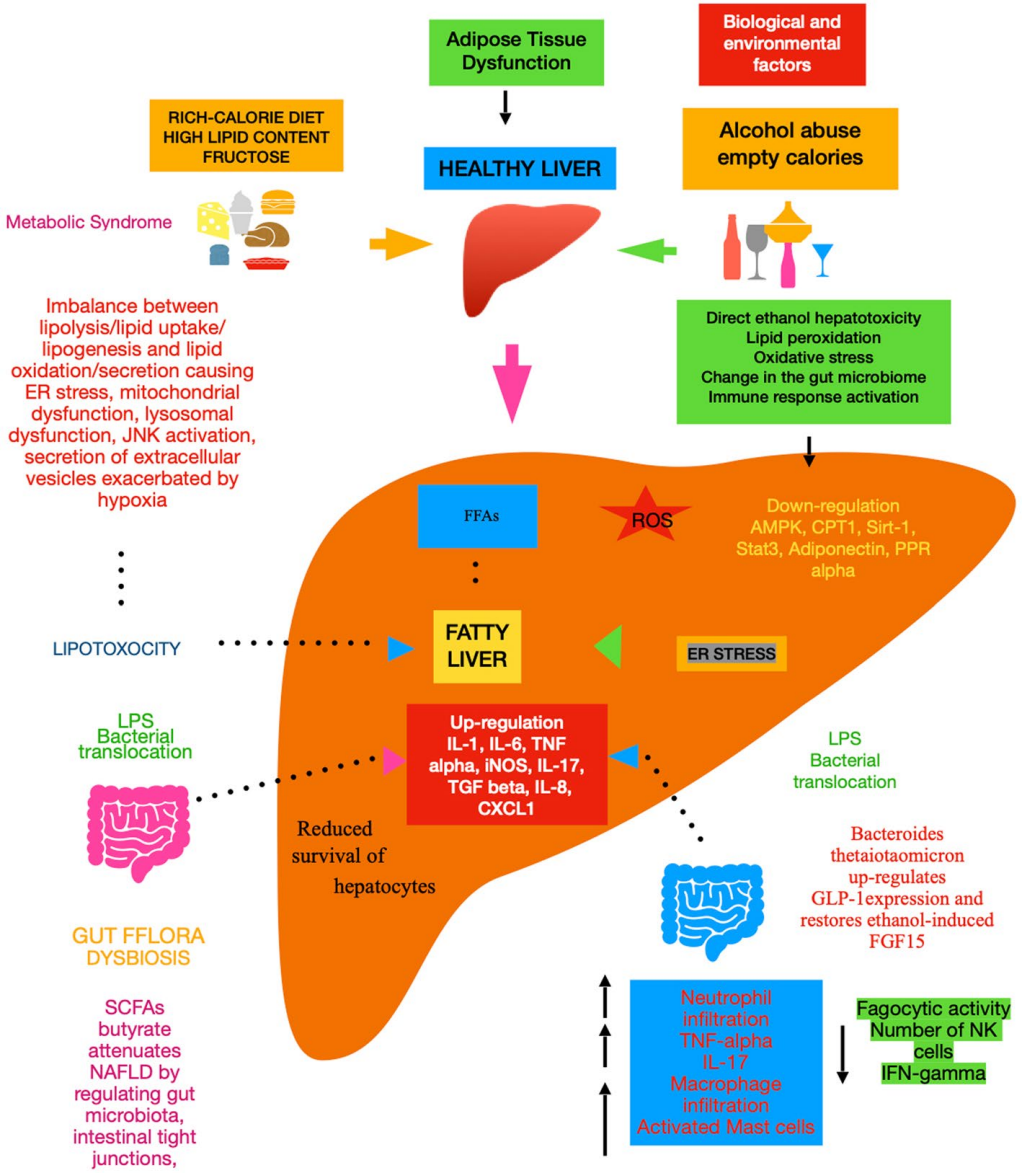
Common and different mechanisms of ALD and NAFLD/MASLD. Abbreviations: ER, endoplasmic reticulum; JNK, c-Jun N-terminal kinase; FFAs, free fatty acids; AMPK, AMP-activated protein kinase; ROS, reactive oxygen species; CPT1, carnitine palmitoyltransferase I; Sirt, sirtuin; Stat3, signal transducer and activator of transcription 3; PPR alpha, peroxisome proliferator-activated receptor alpha; IL, interleukin; TNF, tumor necrosis factor; iNOS, inducible nitric oxide synthase; TGF, transforming growth factor; CXCL1, C-X-C motif chemokine ligand 1; GLP, glucagon-like peptide; FGF15, fibroblast growth factor 15; IFN, interferon; NK, natural killer

The default position in categorizing the two initially discussed forms of hepatic steatosis and the successive spectra is that there is no relationship between the two entities. In this review, we try to test this null (i.e., zero) hypothesis by examining the biological mechanisms that explain these diseases, drawing some conclusions from our current approach to elicit the potential of new interventions (Fig. 1).

## Characteristics of NAFLD and ALD

Zeroing in on the main factors able to clinically differentiate NAFLD from ALD is essential. Alcohol consumption can be evaluated as alcohol consumption (e.g., drinks per week) or as a lifetime measure (e.g., alcohol dependence). One of the most common criteria for evaluating excess alcohol consumption is drinking more than 21 drinks per week for men and more than 14 drinks per week for women [21]. Indeed, the two liver diseases often coexist. Among 1,216 patients with alcohol-associated acute-on-chronic liver failure (ACFL), overweight/obese status and dyslipidemia increase the severity of alcohol-associated ACLF, and a high BMI augments short-term mortality [22]. Paradoxically, a meta-analysis of 43,175 subjects showed that modest alcohol consumption decreases the risk of NAFLD [23].

With respect to risk factors, it is necessary to stress the differences between the two main entities. The relative risk of ALD is greater in women for any given amount of alcohol intake [24]. Conversely, NAFLD has a greater prevalence in men. Compared with men, women of fertile age are at reduced risk of NAFLD, whereas after menopause, women have a comparable prevalence of NAFLD to men [25]. The proportion of Hispanic and White/Caucasian patients with severe alcoholic hepatitis was similar but was lower for African/American subjects [26]. These racial differences are likely due to genetic differences in the enzymes involved in alcohol metabolism. Accordingly, genome-wide association studies.

GWASs of alcohol consumption in European Americans and African Americans have identified significant associations with the well-known functional locus alcohol dehydrogenase 1B (ADH1B) [27]. Very recently, authors, carrying out a GWAS meta-analysis of imaging and diagnostic code-measured NAFLD across diverse ancestries, identified NAFLD-associated variants at torsin family 1 member B, fat mass and obesity associated, cordon-bleu WH2 repeat protein like 1/growth factor receptor-bound protein 14, insulin receptor, sterol regulatory element-binding transcription factor 1 and patatin-like phospholipase domain-containing protein 2, as well as validated NAFLD-associated variants at patatin-like phospholipase domain-containing protein 3, transmembrane 6 superfamily 2, apolipoprotein E, glucokinase regulator, tribbles homolog 1, glycerol-3-phosphate acyltransferase, mitochondrial amidoxime-reducing component 1 (MARC1), microsomal triglyceride transfer protein large subunit*,* transmembrane channel like 4/membrane-bound O-acyltransferase domain containing 7 and receptor-type tyrosine-protein phosphatase δ, and, surprisingly, ADH1B [28]. It should be stressed that the aforementioned variants in MARC1 are also novel genetic risk factors for ALD [29].

Now, we attempt to determine the similarities but also differences in histopathology features. Macrovesicular hepatic steatosis is the main feature of NAFLD and is the initial and most common consequence of excessive alcohol consumption due to the impaired intracellular redox potential that leads to the accumulation of lipids within liver cells. In addition, small fat droplets collect around hepatocytes in areas filled with venules, proceeding toward the portal tracts. The first form of ALD, i.e., pure AFLD, may not be a benign condition every time. In fact, when retrospectively analyzing patients with a histologic diagnosis of AFLD without alcoholic steatohepatitis, as many as 18% progressed to fibrosis or cirrhosis when followed for a median of ten years. However, it is necessary to stress that the severe forms were linked to continuing alcohol consumption of more than 40 units per week at follow-up, while in the other patients, consumption was unknown [30], leaving doubts whether the progression could be due to initial liver fat storage. Thus, additional evidence is needed to determine whether hepatic steatosis plays a role in the onset and progression of advanced liver disease.

Eosinophilic fibrillar material (Mallory hyaline or Mallory-Denk bodies that are “damaged intermediate filaments” within liver cells) produces swollen hepatocytes. Ballooned cells are typically two to three times the size of adjacent hepatocytes. This histological feature distinguishes steatohepatitis from simple steatosis. Severe lobular infiltration of polymorphonuclear leukocytes (neutrophils) is abundant in alcoholic steatohepatitis, unlike in other types of hepatitis, such as viral hepatitis, where mononuclear cells are restricted around portal triads. The final stage of ALD is alcoholic cirrhosis, characterized by the deposition of collagen that typically starts around the terminal hepatic vein (perivenular fibrosis) and along the sinusoids, leading to a peculiar “chickenwire” pattern of fibrosis [31]. Some authors insist that individual cases of ALD can be differentiated from NAFLD by the presence of sclerosing hyaline necrosis, fibro-obliterative and inflammatory lesions of the outflow veins, alcoholic foamy degeneration, and acute cholestasis [32]. According to other pathologists, hepatic steatosis is more common in NAFLD patients than in ALD patients. Conversely, inflammatory cell infiltration is more pronounced in ALD than in NAFLD. Furthermore, venous or perivenular fibrosis, phlebosclerosis, and (less commonly) lymphocytic phlebitis are more common in ALD patients than in NAFLD patients [33]. Indeed, upon careful examination of silver impregnation histology, the progressive form of ALD, i.e., steatohepatitis, could be detected as lattice fibrosis in NASH and solid fibrosis in ALD. In addition to previous findings, megamitochondria, bile stasis, hemosiderin deposition, vacuolic nuclei, and lipogranuromas were less common in ALDs [34]. Overall, there are subtle differences between NAFLD and ALD, even though they are more advanced (Table 1). For this reason, distinguishing one from the other is difficult, according to the classic study by Ludwig [35].

**Table 1:** Main features of alcoholic and nonalcoholic fatty liver disease at histology

Alcoholic Liver Disease	Non Alcoholic Fatty Liver Disease
Macrovescicular steatosis is largely represented	Macrovescicular steatosis is less represented
Mallory hyaline is recurrent	Mallory hyaline is not occurring often
Swollen hepatocytes/Ballooned cells are more frequent	Swollen hepatocytes/Ballooned cells are less present
Lobular infiltration of polymorphonuclear leukocytes (neutrophils) is severe	Usually, there is mild lobular infiltration with foci of mononuclear cell clusters, and occasional eosinophils or neutrophils.
Inflammatory cell infiltration is more pronounced	Inflammatory cell infiltration is less marked
Perivenular fibrosis with the chicken wire” pattern of fibrosis is common	Fibrosis typically begins in zone 3 with the characteristic pericellular “chicken wire” pattern
Fibro-obliterative/inflammatory lesions of the outflow veins, alcoholic foamy degeneration are present	Fibro-obliterative lesions are not constant and foamy degeneration is rare
Acute cholestasis is often present	Intrahepatic cholestasis is associated with more advanced histological impairment
Phlebosclerosis, and (less commonly) lymphocytic phlebitis are present	Phlebosclerosis is rare
There is solid fibrosis	There is lattice fibrosis
Megamitochondria, bile stasis, hemosiderin deposition, vacuolic nuclei, and lipogranuroma are scarcely represented	Megamitochondria, bile stasis, hemosiderin deposition, vacuolic nuclei, and lipogranuroma are more often represented
Bridging necrosis is frequent	Bridging necrosis is rare
Fibrosis/cirrhosis is more frequent	Fibrosis/cirrhosis is less frequent

The features at histology overlap, and it is not easy to clearly separate the two entities

## Common molecular events

### Lipid metabolism alterations

As previously described, the initial process characterizing hepatic steatosis consists of lipid droplets that are enriched in saturated triglycerides and result in the typical histological pattern of steatosis common to both NAFLD and AFLD [36]. Liver fat storage originates from a mismatch of fatty acid uptake (either by nutrients or from adipose tissue), fatty acid synthesis, fatty acid oxidation, or the export of lipids from the liver through effects on apolipoprotein B and microsomal transport protein [37]. Zeroing on lipid synthesis, sterol regulatory element-binding protein (SREBP) 1 plays a central role in NAFLD but not only [38]. In fact, activation of SREBP-1 by ethanol feeding was associated with increased expression of hepatic lipogenic genes as well as the accumulation of triglycerides in the liver in both the rat H4IIEC3 and McA-RH7777 hepatoma cell lines [39]. However, downstream signaling events involving transcription factor-regulating genes involved in lipid synthesis and the endoplasmic reticulum (ER) stress response are present in both NAFLD and ALD in the context of fatty acid oxidation mediated by AMP-activated protein kinase (AMPK), adiponectin, and peroxisome proliferator-activated receptors (PPARs) [40]. Interestingly, findings suggest that activation of this latter pathway may improve NAFLD due to the intrinsic link between NAFLD and mitochondrial metabolism and the mechanisms by which mitochondrial dysfunctions contribute to NAFLD progression [41].

### Redox imbalance

Importantly, another central effect of ethanol on lipids is to activate (e.g., via ER stress, tumor necrosis factor (TNF)-alpha and/or hepatic PPAR-gamma) de novo lipogenesis (DNL) while concomitantly inhibiting processes that block this response, such as AMP-activated protein kinase (AMPK) and sirtuin (SIRT) 1 [42]. As a significant event, ROS production and oxidative stress in liver cells play pivotal roles in the development of ALD [43]. Confirming the importance of oxidative stress in ALD, the authors showed that Humulus japonicus extract decreases ethanol-induced lipid accumulation, lipogenic protein expression, and cellular oxidative stress in hepatocytes [44]. A further epiphenomenon induced by triglyceride excess and the accumulation of other lipids is the generation of insulin resistance in both NAFLD [45] and AFLD [46].

The key molecular event represented by DNL is also central to NAFLD. DNL was measured isotopically and was significantly associated with liver fatty acid synthase protein content, total steatosis assessed by histology, and the fraction of DNL fatty acids in plasma very low-density lipoprotein-triacylglycerol in 49 patients scheduled for bariatric surgery [47].

### The main role of adipokines

It is well known that adiponectin lowers insulin resistance by decreasing triglyceride content in the muscle and liver in obese mice [48]. In this context, the role of adiponectin, among other mechanisms, needs to be clarified (Table 2). In fact, previous findings have shown that altered adiponectin production in adipose tissue and impaired expression of hepatic adiponectin receptors are associated with the development of AFLD in several rodent models [49]. Similarly, adiponectin generally predicts steatosis grade and the severity of NAFLD, although to what extent this is a direct effect or related to the presence of more severe insulin resistance or obesity remains to be addressed [50]. In vitro and in vivo studies have shown that another crucial adipokine, gremlin-1, promotes hepatic ER stress through the impairment of autophagy, which we briefly focus on, ultimately causing hepatic steatosis in the obese state [51].

**Table 2:** Signaling events of alcoholic liver disease and nonalcoholic fatty liver disease

Alcoholic Liver Disease	Non Alcoholic Liver Disease
SCFAs are decreased	Fecal SCFAs are increased
Secondary BAs are high acting on farnesoid X receptor	BAs binds to the farnesoid X receptor increasing inflammation and fibrosis
Lactobacillaceae correlates with postprandial peripheral ethanol	It is evident a reduction in the abundance of *Ruminococcus gnavus* and *Bifidobacterium longum subsp infantis*
*Klebsiella pneumoniae* strain W14 bears the higher ethanol production	Some forms of NAFLD are due to endogenous production of ethanol
Chronic intake of alcohol beyond provoking ROS formation raises the multiplication of intestinal bacteria and changes the gut permeability	High-alcohol-producing *Klebsiella pneumoniae* is associated with up to 60% of individuals with NAFLD in a Chinese cohort.
Pathogenic species of bacteria results in a fast replenishment of the marginal zone of the spleen	The spleen has been recognized to take a great part in lipid metabolism via the mononuclear phagocytic system
Functional hyposplenism is present	There is a trend in increased spleen volume
K18 is the main component of Mallory-Denk bodies, which are a hallmark of alcoholic steatohepatitis	K18 fragments have been proven to be associated with NASH
Serum TPS, mirroring K18, is frequently increased in alcoholics and may be a marker of alcoholic hepatitis	TPS is a better marker for differentiating NASH from simple fatty liver
Ethanol-induced oxidative stress directly downregulates NAD + levels, increases SIRT1 nucleocytoplasmic shuttling, and ultimately inhibits SIRT1 gene and protein expression levels in the liver	SIRT1 activator E1231, alleviated NAFLD induced in C57BL/6J mice fed a high-fat and high-cholesterol diet and improved liver injury by regulating the SIRT1-AMPK alpha pathway
SIRT 4 is strongly associated with heavy drinker	Low circulating levels of SIRT4 are found in obese patients with NAFLD
There is a close relationship between SIRT7 and age-related processes	SIRT7 is linked to age-related processes
Impaired expression of hepatic adiponectin receptors are associated with AFLD	Adiponectin predicts steatosis grade and the severity of NAFLD

There are more common than dissimilar mechanisms involved

*Abbreviations*: *BA* biliary acid, *TPS* polypeptide-specific antigen, *K18* karykeratin 18, *ROS* reactive oxygen species, *SCFAs* short-chain fatty acids, *SIRT* sirtuin, *AFLD* alcoholic fatty liver disease, *NAFLD* nonalcoholic fatty liver disease, *NASH* nonalcoholic steatohepatitis, *NAD* nicotinamide adenine dinucleotide, *AMPK* adenosine monophosphate-activated protein kinase

### Cellular senescence

It is necessary to stress which molecular processes could be involved in the pathogenesis of ALD and NAFLD, in addition to canonical insulin resistance, mitochondrial dysfunction, and adipokine imbalance. Cellular senescence, better defined as a stress-responsive program, limits the proliferation of damaged cells by inducing telomere shortening, enlargement of the nuclear area and genomic and mitochondrial DNA damage, ultimately leading to irreversible cell cycle arrest and the secretion of proinflammatory cytokines. Accordingly, dysfunctional lysosomes, a hallmark of cellular senescence, contribute to lipid droplet accumulation in fatty liver disease [52]. Recent results highlight that plasminogen activator inhibitor-1 and early growth response protein 1 are important regulators of lipopolysaccharide-induced cellular senescence and alcoholic hepatitis [53]. Another fascinating study showed that a novel factor, zinc‐finger protein 281, is a driver of hepatocyte senescence during ALD by reducing hexokinase II-stabilized PTEN-induced kinase 1/Parkin-mediated mitophagy [54]. Returning to lysosomes, chronic ethanol consumption disrupts hepatic biogenesis and inhibits the ubiquitin‒proteasome system by impeding hepatic proteasome activity [55]. In line with previous results, several human and animal studies have shown the involvement of senescence in determining NAFLD [56]. Very recently, restoration of lysosomal acidification was shown to rescue autophagy, which shares the same characteristics as senescence and metabolic dysfunction in NAFLD [57]. This field of research could help discover novel therapeutic targets for halting NAFLD progression.

## Main pathogenetic mechanisms of ALD

In addition to the downstream molecular events of ALD, ethanol oxidative metabolism influences intracellular signaling pathways and disrupts the transcriptional control of several genes, leading to fat accumulation and fibrogenesis [58]. Specifically, the role of microsomal ethanol oxidizing system, i.e., P4502E1 is central [59]. In parallel with NAFLD, there was also a marked increase in hepatic CYP2E1 activity in obese T2DM patients, one of the most common risk factors for NAFLD, as assessed by chlorzoxazone administration [60]. Furthermore, alcohol and its metabolites initiate and aggravate inflammatory conditions by sensitizing immune cells to stimulation and by activating innate immune pathways [61]. By decreasing the production of proinflammatory cytokines, chronic exposure to ethanol leads to the translocation of LPS from the gut, activating Kupffer cells through Toll-like receptor 4 (TLR4) and overexpressing IL-1 and TNF-alpha. These cytokines contribute to hepatocyte dysfunction and programmed cell death, as well as to the activation of hepatic stellate cells (HSCs), which generate extracellular matrix proteins leading to fibrosis development [62].

IL-6 plays dual roles in the pathogenesis of ALD by not only favoring inflammation and injury but also promoting liver regeneration [63]. In fact, IL-6 deficiency in mice has been shown to protect against ethanol-induced oxidative stress and mitochondrial dysfunction in hepatocytes via the induction of metallothionein protein expression [64]. This occurs at least in the early phase of ALD. Conversely, in alcoholic liver cirrhosis, increased serum levels and spontaneous or induced production of IL-6 by peripheral blood monoclonal cells have been found [65]. IL-8, also known as CXCL8, and CXCL1 promote liver inflammation and injury by stimulating neutrophil infiltration [66]. After the progression of ALD, mechanistically, acetaldehyde, a major toxic metabolite of ethanol, causes adduct formation, representing one of the principal causes of the fibrogenic and mutagenic effects of alcohol in the liver [67]. Malondialdehyde and acetaldehyde react with proteins in a synergistic manner and form hybrid protein adducts, playing an important role in the pathogenesis of alcoholic liver injury [68]. Finally, ALD is associated with the generation of IgAs and IgGs against acetaldehyde-derived antigens [69]. These last molecular initiation events fundamentally differ from those of NAFLD.

## Inflammation and NAFLD, recently renamed MASLD

The mechanisms underlying MAFLD involve intertwined processes involving lipotoxicity and infiltration of proinflammatory cells with cytokine/chemokine overexpression, resulting in HSC activation and fibrogenesis [70]. However, the interaction between mitochondrial dysfunction and insulin resistance has also been typically described in the context of NAFLD, and this mechanism is being increasingly regarded as pivotal for leading to hepatic steatosis [71]. Dysregulated mitochondrial oxidative metabolism mediates inflammation and liver fibrosis [72]. Coming back to inflammation/immune system involvement as a link between excess fat storage in the liver and NAFLD progression, authors used an animal model of NASH and found increased serum concentrations of TNF-alpha, TLR4 and CD14 in male F344 rats that were fed a choline-deficient L-amino-acid-defined diet [73]. Similarly, in 52 obese patients, a remarkable increase in the expression of TNF-alpha mRNA was found in hepatic tissue and peripheral fat in patients with NASH [74]. The expression of IL-6, the main proinflammatory cytokine, was markedly increased in the livers of patients with NASH compared to patients with simple steatosis or normal biopsies, confirming that hepatic IL-6 expression plays an important role in NAFLD progression, as well as in systemic insulin resistance, correlating plasma IL-6 levels with the degree of systemic insulin resistance [75]. IL-10(-/-) mice are resistant to steatosis and hepatocellular damage induced by ethanol or HFD feeding. This is due to the increase in inflammation-associated hepatic IL-6/STAT3 activation, which subsequently downregulates lipogenic genes but upregulates fatty acid oxidation-associated genes in the liver [76].

Similarly, in an animal model of HFD-induced NAFLD, the inhibition of IL-10 results in increased expression of inflammatory cytokines, worsening of insulin signaling and activation of gluconeogenic and lipidogenic pathways [77]. As previously mentioned, activated IL-8 mediates neutrophil infiltration, a pivotal process in the pathogenesis of ALD. Furthermore, high IL-8 levels might reflect the severity of ALD [78]. Surprisingly, increased concentrations of IL-8, a consequence of hepatic gene expression, were found in 97 patients with biopsy-proven NAFLD, lending credence to such chemokines as ideal surrogate serum biomarkers for the severity of hepatic fibrosis [79]. Another attractive cytokine involved in this very common liver disease, MASLD, is IL-17. Clinical trials have shown that inhibiting IL-17A could contribute to the improvement of MAFLD patients with psoriasis [80]. Interestingly, palmitic acid-induced cellular stress in liver epithelial cells enhances the expression of IL-32 and CCL20, contributing to increased expression of these fibrosis-driving molecules in MASLD [81].

## High-fat diet/T2DM induces NAFLD/MASLD

Canonically, fatty liver is generally connected with a HFD, insulin resistance and obesity.

Various downstream signaling events, including mitochondrial dysfunction, endoplasmic reticulum stress, derangement of autophagy, cellular apoptosis, gut flora dysbiosis, dysregulation of microRNAs, and genetic/epigenetic risk factors, and an increase in inflammatory responses, have been found to account for this liver damage [82]. Progress over the last decade has been substantial in that the role of adipose tissue inflammation and the gut microbiome (gastrointestinal tract emerging as critical drivers of inflammation) have evolved as crucial players in the pathogenesis of NAFLD, suggesting multiple parallel hits based on mechanistic insights gained from animal models and descriptive clinical trials [83]. First, there is a complex two-way relationship between the progression of NAFLD and the development of T2DM [84]. A central pathogenic mechanism of NAFLD is insulin resistance in the liver and extrahepatic tissues such as skeletal muscle, beyond the already emphasized adipose tissue. Specifically, the molecular initiator of hepatic insulin resistance is protein kinase C translocated to the membrane compartment from the cytosol, causing impaired activation of the insulin receptor substrate phosphatidylinositol-3 kinase [85]. Recent data suggest that decreased mitochondrial content in the muscle of insulin-resistant offspring may be due in part to reductions in lipoprotein lipase expression in skeletal muscle resulting in decreased PPAR-delta activation [86].

## Therapeutic approaches for ALD and NAFLD/MAFLD

Long-term abstinence is currently the main treatment for ALD [87]. As a further approach, nutritional support is recommended for patients by providing them with high-protein, low-fat diets and by balancing the levels of vitamin B, C, K, and folic acid [88].

The authors found that an association between silymarin and S-adenosyl-L-methionine, recently introduced to the market, seems to be promising in such cases of ALD [89]. An interesting study of Gclm KO mice revealed a novel mechanism that protects against liver steatosis via an oxidative stress adaptive response that activates the AMPK pathway. Accordingly, the authors suggested that redox activation of AMPK may represent a new therapeutic strategy for preventing ALD. Oxidative stress and associated decreases in glutathione levels are known to play central roles in ALD [90]. Lipin 1 is a key regulator of hepatic lipid metabolism and a downstream target of miR-203. Recent results suggested that overexpression of miR-203 inhibits liver lipid accumulation and the progression of AFLD by targeting lipin1 [91]. IL-22 has antiapoptotic, antifibrotic, antioxidative, antibacterial and regenerative effects on protecting against liver injury in many preclinical models, including several recently developed models, such as chronic-plus-binge ethanol feeding [92].

Returning to the gut flora, the colonic microbiome is impaired in alcoholism patients, in which the median abundance of Bacteroidetes is lower and that of Proteobacteria is greater [93]. Thus, both intestinal dysbiosis and disturbed integrity of the intestinal barrier play a role in the promotion of inflammatory liver damage in chronic excessive use of alcohol. Prebiotics, probiotics, postbiotics, and symbiotics could be useful therapeutic interventions for the treatment of ALD [94]. Oral ethanol supplementation diminishes the intestinal abundance of *A. muciniphila* in both mice and humans, but this effect can be reversed in mice with experimental ALD. In fact, *A. muciniphila* promotes intestinal barrier integrity and ameliorates experimental ALD [95]. Oral supplementation with living *Bacteroides thetaiotaomicron*, which upregulates glucagon-like peptide expression and restores ethanol-induced fibroblast growth factor 15 downregulation, was associated with restored intestinal homeostasis and ameliorated experimental ALD [96]. As various cytokines are upregulated in the liver and increased in the serum of patients with mild/moderate and severe forms of ALD, they may be valid targets for curing this disease [97]. Authors have shown that mice deficient in interferon regulator factor 3 or TLR4 expression are protected from alcohol-induced liver steatosis, inflammation and hepatocyte injury, suggesting that inflammasome and caspase-1 activation occur in ALD and that IL-1 significantly contributes to both steatosis and inflammation in the liver in ALD [98]. The finding that the inflammatory cascade plays a central role in the pathophysiology of ALD was further confirmed by a classical clinical investigation that showed that the anti-TNF monoclonal antibody infliximab resulted in an early, though not significant, decrease in the plasma levels of the proinflammatory cytokines IL-1beta, IL-6, IL-8, and interferon-gamma, whereas the plasma levels of TNF-alpha remained near the sensitivity limit of the assay throughout the treatment course in ten out of the 12 patients [99].

The mainstream NAFLD therapy consists of intensive lifestyle modifications, in the sense of a proper diet and exercise [100], beyond weight control [101]. Indeed, there are many drugs in the pipeline that are considered good candidates for curing NAFLD/NASH, as reviewed previously [102]. In addition to the key role of cytokines, IL-22 activates the JAK1/signal transducer and activator of transcription 3, c-Jun N-terminal kinase and extracellular-signal regulated kinase pathways, leading to subsequent regulation of the expression of genes involved in inflammation, metabolism, tissue repair, and regeneration, thus creating therapeutic opportunities for alleviating NAFLD [103]. Another protective effect on liver injury is provided by the noncoding gene miR-29a due to its ability to regulate epigenetic activity, mitochondrial homeostasis and immune modulation, which could have therapeutic applications in NAFLD [104]. Finally, the findings of these studies suggest that sodium butyrate and *Clostridium butyricum* are potential adjuvant treatment strategies for T2DM-induced NAFLD [105].

## The gut microbiome: a key regulator of host physiology

The gut microbiome plays a central role in modifying blood lipid levels, independent of age, sex, and host genetics [106]. On the other hand, lipid metabolism is instrumental to the onset of hepatic steatosis, as previously emphasized. The gut microbiota transforms and synthesizes lipids and breaks down dietary lipids to generate secondary metabolites with host modulatory properties. Previously, lipids have largely been considered to play structural roles, such as in cell membranes, or viewed as metabolic fuel, but recent research has led to an increased appreciation of their signaling activities [107]. In keeping with this emerging role, some endogenous bioactive lipids produced from arachidonic acid and other polyunsaturated fatty acids exert their biological activity via specific receptors by triggering distinct signal transduction pathways [108].

An increasing body of evidence points to a bidirectional interaction between the gut and the liver mediated by the microbiome. On this basis, chronic intake of alcohol beyond provoking ROS formation increases the multiplication of intestinal bacteria and changes the gut permeability to food antigens, commensal or pathogenic bacteria and bacterial components, ultimately increasing endotoxins in the portal circulation and activating Kupffer cells through Toll-like receptor pathways [109]. In contrast, there is a major group of microbial metabolites, i.e., short-chain fatty acids (SCFAs), with favorable effects. They promote the growth of intestinal epithelial cells and strengthen intestinal tight junctions, ultimately maintaining the integrity of the gut barrier. Moreover, these products regulate peripheral innate immune responses [110]. There is wide consensus on the finding that SCFA levels decrease with alcohol consumption [111]. Accordingly, linolenic acid, by shaping the microbial metabolome, could be used for the treatment of ALD [112].

Conversely, data on SCFAs in NAFLD patients are controversial. However, for some authors, higher fecal SCFA levels and higher abundance levels of SCFA-producing bacteria were found in NAFLD patients [113]; moreover, fresh evidence shows that circulating SCFA levels are lower in NAFLD patients and negatively correlated with the severity of T2DM-associated NAFLD [114]. The likely mechanism linking SCFAs to the progression of NAFLD is based on the finding that these SCFAs are negatively related to TNF-alpha [115].

In addition to the previously mentioned events, intestinal bacteria transform primary bile acids (BAs) to secondary BAs, which can modify the gut barrier. In this context, alcohol abuse is associated with a significant increase in the secondary formation of BAs, which are implicated in the pathogenesis of ALD [116, 117]. Notably, BA binds to the farnesoid X receptor, which is critically involved in many molecular processes responsible for maintaining glucose and lipid homeostasis. Alterations to these pathways can also lead to dysregulation of energy balance and increased inflammation and fibrosis in NAFLD patients [118].

Considering the phylogenetic profiles of the gut microbiota of NAFLD patients, we should note that studies partially converge on the class, family and genus levels of the involved bacteria. Specifically, low abundances of *Faecalibacterium*, *Bacteroides* and *Prevotella* and high abundances of *Gemmiger* were associated specifically with the degree of inflammation, ballooning, and stage of fibrosis, even after taking other cofactors into account, in 57 NAFLD patients [119]. Alternatively, the intestinal bacterial microbiota in NAFLD patients with coronary artery disease showed an increase in the abundance of *Copococcus* and *Veillonella* and a reduction in the abundance of *Ruminococcus gnavus* and *Bifidobacterium longum subsp. infantis,* in addition to *Parabacteroides, Bacteroides fragilis* and *Bacteroides dorei* [120].

Interestingly, in this field of research, alcohol produced by some intestinal bacteria is associated with liver damage. The endogenous production of ethanol was confirmed by an interesting preclinical study in which two mutants with different alcohol-producing abilities, W14-*adh* and W14Δ*adh*, were constructed using the clinical high alcohol-producing *Klebsiella pneumoniae* strain W14 as a parent. Mice in the W14-*adh* group, which had the highest ethanol production, exhibited the most severe liver damage, characterized by ROS accumulation, likely as a consequence of mitochondrial dysfunction [121]. Data from an intervention study of ten NAFLD patients in which ethanol was measured in the portal vein blood of patients infused with a selective alcohol dehydrogenase inhibitor before a mixed meal test was performed showed that the first-pass effect obscures the level of endogenous ethanol production, suggesting that microbial ethanol could be considered to be involved in the pathogenesis of NAFLD. Furthermore, Lactobacillaceae correlated with postprandial peripheral ethanol concentrations, findings that were prospectively obtained [122]. These results suggest that, at least in some cases of NAFLD, an alteration in the gut microbiome drives this pathological condition due to excess endogenous alcohol production. Moreover, in a Chinese cohort, investigators discovered that high-alcohol-producing *Klebsiella pneumoniae* (HiAlc Kpn) is associated with up to 60% of individuals with NAFLD. Nevertheless, the same group found that the oral gavage of clinical isolates of HiAlc Kpn into mice induced NAFLD. Finally, selective elimination of the HiAlc Kpn strain before focal microbiome transplantation prevents NAFLD in recipient mice [123]. Other data suggest that phage therapy targeting the gut microbiota is an alternative to antibiotics, with potential efficacy and safety, at least in HiAlc *Kpn*-induced NAFLD [124]. In NASH patients, an increased abundance of alcohol-producing bacteria corresponds to an elevated blood ethanol concentration, suggesting a role for alcohol-producing microbiota in the pathogenesis of NASH [125]. However, the main role of the microbiome in NAFLD has not been fully elucidated.

## Spleen and microbiome/cytokeratins

The spleen has been recognized to play a great role in lipid metabolism, likely due to its natural immune function, i.e., via the mononuclear phagocytic system [126]. An increase in the levels of total cholesterol and low-density lipoprotein was observed in splenectomized patients [127]. At this point, we should consider that there is a reciprocal link in the spleen-gut-microbiota axis. In fact, data suggest that splenectomy leads to an abnormal composition of the gut microbiota, impacting SCFA levels [128]. On the other hand, the presence of a greater representation of pathogenic species of bacteria results in rapid replenishment of the marginal zone of the spleen, thus impacting the maturation of the spleen and its complete function [129]. Notably, the authors demonstrated that after 4 days of antibiotic treatment, which eliminated specific families of the *Bacteroidetes, Firmicutes, Tenericutes and Actinobacteria* phyla in the intestine, there was a consequent decrease in the levels of multiple pattern recognition receptor (PRR) ligands. A reduction in the serum concentration of PRR ligands led to a decrease in the number of splenic Ly6Chigh monocytes, which acquired an immature phenotype characterized by decreased inflammatory cytokine levels and increased phagocytic ability [130]. It has long been known that functional hyposplenism occurs in ALD [131], but the spleen-liver axis has gained much attention in recent years concerning NAFLD, as this phenomenon has not been detected in diagnostic versants [132].

An increasingly important topic is the role of cytokeratins in ALD and NAFLD. First, by immunoelectron microscopy, several authors have identified a direct linkage between lipid droplet-binding proteins and intermediate-sized filament cytokeratins (K) 8 and 18 [133]. K18 represents the major intermediate filament protein in liver cells [134] but is also the main component of Mallory-Denk bodies, which are a hallmark of alcoholic steatohepatitis [135]. In NAFLD, K18 fragments have been proven to be associated with NASH, although with reduced sensitivity but good specificity (66% and 82%, respectively) [136]. A previous study showed that polypeptide-specific antigen (TPS), a serological marker of K18 that is widely used as a marker for various cancers, is a better marker for differentiating NASH from simple fatty liver [137]. The serum TPS is frequently increased in alcoholic patients and may be a marker of alcoholic hepatitis [138].

## Sirtuins: as therapeutic targets

SIRTs are effective at modulating energy metabolism by intervening in oxidative stress and mitochondrial homeostasis, regulating the inflammatory response, activating autophagy and necroptosis and acting on lipid metabolism in ALD. Its actions include mediating multiple signal transduction pathways, such as the mTOR, AMPK, PPAR alpha and gamma, LKB1, PGC-1 alpha, FoxO1/3a, Nrf2/p62, TFEB, RIPK1/3, HMGB1, NFATc4, NF-κB, TLR4, NLRP3, P2X7R, MAPK, TGF1β/Smads, Wnt/β-catenin, Lipin1, and SREBP1 pathways [139].

One of the most common SIRTs involved is SIRT1 because ethanol-induced oxidative stress directly downregulates NAD + levels, increases SIRT1 nucleocytoplasmic shuttling, and ultimately inhibits SIRT1 gene and protein expression in the liver [140].

A recent study illustrated that the SIRT1 activator E1231, a piperazine 1,4-diamide compound, alleviated NAFLD induced in C57BL/6 J mice fed a high-fat and high-cholesterol diet and improved liver injury by regulating the SIRT1-AMPK alpha pathway, suggesting that this compound is a promising candidate compound for NAFLD treatment [141].

Treatment of C2C12 myocytes and HepG2 cells with patchouli alcohol augmented AMPK phosphorylation and SIRT1 expression in a dose-dependent manner, attenuating skeletal muscle insulin resistance and hepatic steatosis in high-fat diet-fed mice [142].

Interestingly, SIRT4 is an important regulator of lipid homeostasis, and malonyl CoA decarboxylase has been identified as a SIRT4 target [143].

There are conflicting results related to the role of SIRT4 in the development of atherosclerosis, which is strongly associated with NAFLD [144] and heavy alcohol consumption [145]. SIRT4 overexpression suppressed the PI3K/Akt/NF‑κB pathway by inhibiting PI3K phosphorylation and phosphorylated (p)‑Akt and inhibiting the expression of inflammatory cytokines in oxidized low-density lipoprotein‑induced human umbilical vein endothelial cells [146]. In contrast with the findings of previous studies, other authors have shown that SIRT4 deficiency exacerbates inflammation and promotes the development of atherosclerosis, activating the phosphorylation of NF-κB [147]. Consistent with these data, low circulating levels of SIRT4 were found in obese patients with NAFLD and early atherosclerosis, likely mirroring its reduced mitochondrial expression in an attempt to increase fat oxidative capacity and mitochondrial function in the liver and muscle [148].

Nevertheless, researchers revealed that treating hepatocytes with valdecoxib increases AMPK phosphorylation and SIRT6 expression in human primary hepatocytes and ameliorates hepatic lipid accumulation and lipogenic protein expression in HFD-fed mice, thus attenuating hepatic steatosis under hyperlipidemic conditions [149]. Several reports have substantiated the close relationship between SIRT7 and age-related processes [150]. Earlier findings suggest that musclin can suppress palmitate-induced ER stress by upregulating SIRT7 and autophagy signaling, thereby alleviating lipid accumulation in primary hepatocytes [151].

## Other common mechanisms

Close examination of the oxidative damage associated with NAFLD revealed that ablation of catalase promoted NAFLD via oxidative stress and mitochondrial dysfunction in diet-induced obese mice. Furthermore, knockdown of catalase expedited cellular lipid accumulation and weakened mitochondrial biogenesis in fatty acid-treated HepG2 cells [152]. Catalase knockout mice were not effective at metabolizing alcohol or inducing hydrogen peroxide clearance and were more prone to alcohol-induced liver injury. Catalase-mediated hydrogen peroxide removal has been found to be an inherent mechanism through which PPAR-alpha maintains the nicotinamide adenine dinucleotide pool [153]. However, preclinical studies concerning catalase favor its role in addition to preclinical studies. Recent in vivo observations have shown that decreased catalase levels, leading to an insufficient antioxidant defense system, are a characteristic of 139 NAFLD patients [154]. In addition, methyl donors, such as S-adenosylmethionine (SAMe) and betaine, may act via the attenuation of alcohol-induced oxidative stress in male C57BL/6 J mice [155]. Moreover, interesting findings highlight the beneficial effect of SAMe as an antisteatotic agent in two in vitro models of fatty liver [156]. Now, we try to explain which mechanisms NAFLD and AFL share on the genetic side. Patatin-like phospholipase domain-containing proteins (PNPLA1-9) are a family of proteins that have diverse lipolytic activities toward diverse substrates, such as triacylglycerols, phospholipids, and retinol esters [157]. Previous data have shown that the PNPLA3 I148M mutation promotes triglyceride accumulation by limiting triglyceride hydrolysis [158]. It has recently been suggested that the rs738409 G allele in PNPLA3, which encodes adiponutrin, is strongly associated with increased liver fat content in different ethnic groups [159]. Genetic variation in PNPLA3 confers susceptibility to NAFLD [160, 161] and is associated with ALD [162, 163].

Substitution of lysine for glutamic acid at residue 167 in transmembrane 6 superfamily member 2 (TM6SF2), located on chromosome 19, is associated with fatty liver disease. The authors found that TM6SF2 acts in the smooth endoplasmic reticulum to promote bulk lipidation of apolipoprotein B-containing lipoproteins, thus preventing fatty liver disease in a study comparing Tm6sf2-/- mice with their wild-type littermates [164].

Because carriers of the *TM6SF2* variant have favorable cardiovascular outcomes, identification of downstream pathways altered by the *TM6SF2* E167K variant could help unravel its noxious role in NAFLD development. Furthermore, this study contributes to the development of new therapeutic approaches due to its cardioprotective effect [165].

In addition, miR-34a, miR-122 and miR-155 are the most involved in the pathogenesis of NAFLD. These three miRNAs have also been implicated in ALD, reinforcing a common disease mechanism between these two entities and the pleiotropic effects of specific miRNAs [166].

## Future directions

First, in patients with T2DM, the new definition of MAFLD increased the prevalence of fatty liver disease by 68.89%, resulting in an overdiagnosis of fatty liver disease, exaggerated mortality, and morbidity [167]. Thus, rigorous surveillance is needed.

Considering the n-3 polyunsaturated fatty acid (PUFA) mechanism, i.e., a reduction in de novo lipogenesis and lipid mobilization from adipose tissue, an increase in mitochondrial fatty acid β-oxidation, a decrease in hepatic inflammation and oxidative stress and a positive impact on the intestinal flora, n-3 PUFAs could represent a promising management tool for ALD [168]. However, twelve months of n-3-PUFA treatment in patients with NAFLD was also associated with a significant decrease in liver fat and beneficial changes in the plasma lipid profile [169].

Interestingly, taking into account the mechanisms by which mechanistic target of rapamycin (mTOR) influences insulin resistance by acting on Foxo1 and lipin1; regulating lipid metabolism via SREBPs; interfering with the intestinal microbiota via TLRs; moderating oxidative stress through PIG3, p53, and JNK; and counteracting autophagy, inflammation, genetic polymorphisms, and epigenetics in NAFLD, the authors have stressed the importance of mTOR as an influencing factor of NAFLD [170]. Nevertheless, targeting the signaling of adipose mTOR, nutritional sensors that regulate lipid metabolism, cell proliferation and autophagy, and adipocyte lipolysis could be a potential approach for improving ALD [171] based on the finding that alcohol consumption changes mTORC1 and mTORC2 activity [172]. Importantly, mTOR inhibitors could play a protective role in coronary artery disease [173], a common comorbidity of NAFLD. Furthermore, a recent study provided evidence of how mitochondrial dysfunction, which plays a pivotal role in the alteration of lipid metabolism beyond inflammation and oxidative stress, could be counteracted by natural and bioactive compounds in the context of MASLD [174]. Nevertheless, it would be interesting to further explore why, in heavy drinkers, ethanol oxidation shortens hepatic lipid metabolism, converting the liver from a lipid burning to a lipid-storing organ. In fact, studies involving rodents chronically fed alcohol have shown that ethanol consumption reduces adipose tissue mass by increasing lipolysis in adipose tissue. In this sense, the FFAs released from adipose tissue are taken up by the liver and esterified into triglycerides, thereby exacerbating fat accumulation in the liver [175]. On the other hand, attractive data indicate that the total amount of triglycerides stored in hepatocytes is not the major determinant of lipotoxicity, having the role of ceramides recently emerged in NASH [176]. Capturing, concerning the similar prevention of ALD and NAFLD, is the following. Cyclic guanosine monophosphate–adenosine monophosphate (GMP-AMP) synthase (cGAS) triggers innate immune responses by producing the secondary messenger cyclic 2′,3′-cGAMP, which binds to and triggers STING, resulting in IRF3 activation. RNA sequence analysis of liver cells from ALD patients revealed that activation of the cGAS-IRF3 pathway was related to the severity of ALD. Therefore, cGAS is central to the pathogenesis of ALD and can be used as a potential therapeutic target for liver protection [177]. Accordingly, a study of liver samples from 98 patients with NAFLD also showed that STING expression in Kupffer cells and monocyte-derived macrophages was closely related to inflammation and fibrosis [178]. Failure of the factors that regulate the cGAS-STING pathway is responsible for ALD/NAFLD. Therefore, the cGAS–STING signaling pathway could be a novel and valuable therapeutic target for NAFLD in the future.

Finally, a recent study suggested that genetic variations in neuregulin (*NRG4)* may give rise to mutant proteins with altered functions and that impaired or enhanced Nrg4 function could be either a risk factor or a protective factor for NAFLD and associated metabolic disorders [179].

## Limitations

Currently, many issues persist, and there are still unknown factors involved in the differential diagnosis of NAFLD and ALD. Although these two diseases share similar mechanistic biochemical events, their epidemiological and clinical characteristics are distinct, and there are significant dissimilarities in the prognoses of both illnesses, with alcoholic fatty liver burdened by a much greater risk of developing liver cirrhosis and liver cancer.

## Conclusions

We have tried to answer the main question of whether NAFLD is a completely different disease from ALD or if NAFLD is a two-stage disease caused by the same treatment. The more we learn, the more we realize how similar NAFLD and ALD truly are, at least from pathogenesis. Indeed, there are some other intriguing aspects that must be reported in interpreting the molecular processes displayed during the onset and progression of NAFLD and ALD that are still obscure, as autophagy is a protective or dangerous mechanism and still others. Scientists should prompt new questions to be asked about the molecular events and up-to-date interpretations, challenging and scrutinizing the old ones.

## Data Availability

Not applicable.
